# Breeding, Biosorption Characteristics, and Mechanism of a Lead-Resistant Strain

**DOI:** 10.3390/toxics11050412

**Published:** 2023-04-26

**Authors:** Lining Bao, Yu Cui, Haiwei Wu, Jingwen Xu, Shuguang Zhu

**Affiliations:** 1 Anhui Institute of Strategic Study on Carbon Dioxide Emissions Peak and Carbon Neutrality in Urban-Rural Development, Anhui Jianzhu University, Hefei 230000, China; yuyi18297322318@gmail.com (Y.C.);; 2 School of Environment and Energy Engineering, Anhui Jianzhu University, Hefei 230000, China; 3 Key Laboratory of Water Pollution Control and Wastewater Reuse in Anhui Province, Anhui Jianzhu University, Hefei 230000, China; 4 Engineering Research Center of Building Energy Efficiency Control and Evaluation, Ministry of Education, Anhui Jianzhu University, Hefei 230000, China

**Keywords:** heavy metal pollution, lead-tolerant strains, cultural character, adsorption characteristics, removal mechanism

## Abstract

To effectively carry out the bioremediation of a Pb^2+^ polluted environment, a lead-tolerant strain named D1 was screened from the activated sludge of a factory in Hefei, and its lead removal in a solution with Pb^2+^ concentration of 200 mg/L could reach 91% under optimal culture conditions. Morphological observation and 16S rRNA gene sequencing were used to identify D1 accurately, and its cultural characteristics and lead removal mechanism were also preliminarily studied. The results showed that the D1 strain was preliminarily identified as the *Sphingobacterium mizutaii* strain. The experiments conducted via orthogonal test showed that the optimal conditions for the growth of strain D1 were pH 7, inoculum volume 6%, 35 °C, and rotational speed 150 r/min. According to the results of scanning electron microscopy and energy spectrum analysis before and after the D1 exposure to lead, it is believed that the lead removal mechanism of D1 is surface adsorption. The Fourier transform infrared spectroscopy (FTIR) results revealed that multiple functional groups on the surface of the bacterial cells are involved in the Pb adsorption process. In conclusion, the D1 strain has excellent application prospects in the bioremediation of lead-contaminated environments.

## 1. Introduction

With the continuous development of industrial production and the massive use of fossil fuels, the problem of heavy metal pollution is becoming increasingly serious [1]. Lead, a highly toxic and non-biodegradable heavy metal, poses a serious threat to human health [2]. It is widely used in industries such as battery manufacturing, printing, and electroplating, and is also released into the environment due to mining activities and other reasons [3,4,5]. Heavy metals in the environment enter the ecosystem through water, soil, and airflow [6,7,8], and are absorbed into the body by plants and animals [9,10], thus causing harm to humans health via the food chain [11,12].

Efforts have been made to combat lead pollution through the development of various treatments, such as adsorption [13], membrane technology [14], precipitation [15], and electrodeposition [16]. However, these methods suffer from drawbacks such as high cost, low efficiency, and the potential for secondary pollution. Microorganisms have great potential in treating wastewater contaminated with heavy metals and recovering these pollutants from mining residues or metallurgical wastewater [17]. Many microorganisms, such as fungi, yeasts, and bacteria, are capable of concentrating heavy metals in aquatic environments [18]. Various studies on bioremediation have described different biological materials that can remove heavy metals at a low cost, eliminating small amounts of toxic metals from industrial wastewater or mining residues [19,20]. The aim of this study is to isolate and screen lead-tolerant strains as an efficient and low-cost material to remove higher concentrations of Pb^2+^. The removal mechanism is analyzed preliminarily. These findings can be effectively applied to the bioremediation of heavy metal contaminants.

## 2. Materials and Methods

### 2.1. Materials

The soil was collected from the residual sludge of a plant in Hefei, Anhui Province, for water treatment. Soil samples were collected in sterile bags and stored in the refrigerator at 4 °C for backup.

In this research, all reagents used for the experiments were of at least analytical reagent grade. All solutions were prepared using ultra-pure water in this work. Pb(NO_3_)_2_ was used as the source of lead. In ultra-pure water, Pb(NO_3_)_2_ was dissolved to prepare a solution of the desired concentration. The pH value of the solution was adjusted by applying NaOH and HCl.

The preparation of the culture medium consisted of dissolving 3 g beef extract, 5 g NaCl, and 10 g peptone dissolved in 1000 mL ultra-pure, adjusting the pH to 7.0–7.2 and autoclaving at 121 °C for 30 min. Beef extract-peptone solid medium was added with 10% agar, the same as the liquid medium.

### 2.2. Isolation and Screening of Lead-Resistant Strains

The experimental soil was dissolved in ultra-pure water. The supernatant was then taken and inoculated in a liquid medium with shaking for 2 days (150 r/min, 30 °C). Then, 0.3 mL of the culture solution was taken and diluted by a 10-fold dilution method to coat on 200 mg/L Pb^2+^ solid medium. Then, the culture was put at 30 °C in a constant temperature incubator with inversion for 24 h. The growth state and characteristics of the colonies were observed. Different forms of single colonies were selected for purification and the pure strains were preserved.

The pure isolated strain was shaken and cultured for 24 h. It was then centrifuged at 8000 r/min for 10 min. The bacterium was collected and washed twice with ultra-pure water. Finally, it was made into a bacterial suspension (1 g/L).

The suspensions were cultured in Pb^2+^ medium (200 mg/L) with shaking for 24 h (30 °C, 150 r/min). The medium without inoculum was used as a blank control. The cultured broth was centrifuged at 8000 r/min for 10 min and the supernatant collected. The supernatant was filtered through a 0.22 μm filter membrane and then acidified with HNO_3_; the concentration of Pb^2+^ was determined using inductively coupled plasma (ICP). To screen the best strain for Pb^2+^ adsorption, the lead removal rates were calculated.

### 2.3. Identification of Screened Strains

After the screened strains were inoculated on a solid medium and cultured in an inverted position in a constant temperature incubator (30 °C) for 24 h, the colony morphology was observed. Bacterial fluids were sent to Qingdao Norson Biotechnology Co., Ltd. (Qingdao, China) for sequencing. NCBI-Blast compared the obtained sequences, and phylogenetic trees were constructed using the MEGA 11.0 software.

### 2.4. Effects of Environmental Factors on the Growth of Screened Strains and Lead Removal Rate

#### 2.4.1. Effect of Medium Types on Strain Growth and Lead Removal Rate

The medium types selected for strain growth include meat peptone starch medium, Czapek’s medium, glycerol medium, beef-protein medium, and LB medium. The initial pH of each medium was 7, and the inoculation amount of the strains was 10%. After being cultured for 24 h (30 °C, 150 r/min), the OD_660_ was measured and the supernatant collected. The method of supernatant collection and Pb^2+^ content determination was the same as 3.2.

#### 2.4.2. Effect of Temperature on Strain Growth and Lead Removal Rate

The bacterial suspension was inoculated into the liquid medium (200 mg/L Pb^2+^, pH7) at 10%. After shaking culture at 20 °C, 25 °C, 30 °C, 35 °C, and 40 °C for 24 h (150 r/min), the OD_660_ was measured and the supernatant was collected, respectively. The supernatant was collected for Pb^2+^ content determination.

#### 2.4.3. Effect of pH Value on Strain Growth and Lead Removal Rate

The liquid medium’s pH with a 200 mg/L lead concentration was adjusted to 5, 6, 7, 8, and 9. The bacterial suspension was inoculated at 10%, and after shaking culture for 24 h (30 °C, 150 r/min), the OD_660_ was measured and the Pb^2+^ content was determined.

#### 2.4.4. Effect of Inoculation Volume on Strain Growth and Lead Removal Rate

The bacterial suspensions were inoculated into the liquid medium (200 mg/L Pb^2+^, pH 7) at 2%, 4%, 6%, 8%, and 10%. After shaking culture at 30 °C, 150 r/min for 24 h, the OD_660_ was measured and the supernatant was collected for Pb^2+^ content determination.

#### 2.4.5. Effect of Rotational Speed on Strain Growth and Lead Removal Rate

The bacterial suspension was inoculated into the liquid medium (200 mg/L Pb^2+^, pH 7) at 10%. After shaking culture at 100, 130, 150, 170, and 200 r/min for 24 h, the OD_660_ was measured and the supernatant was collected for Pb^2+^ content determination.

#### 2.4.6. Effect of Adsorption Time on Strain Growth and Lead Removal Rate

The bacterial suspension was inoculated into liquid medium (200 mg/L Pb^2+^, pH 7) at 10%. The shaking culture was conducted at 30 °C and 150 r/min. The OD_660_ and Pb^2+^ concentration were measured every 2 h to investigate the effect of adsorption time on strain growth and lead removal rate.

### 2.5. Orthogonal Test

Based on the single-factor experiments, temperature, pH value, inoculum volume, and rotational speed were selected as the main factors. A 4-factor, 3-level L_9_(3^4^) orthogonal test was conducted with OD_660_ as the optimization target. The factor level table of the orthogonal test is shown in Table 1.

### 2.6. SEM and EDS Analysis

The bacterial suspension was inoculated into the liquid medium (100 mg/L Pb^2+^, pH 7) at 6%. After shaking culture at 150 r/min for 24 h, the bacteria mass was collected and fixed with 2.5% glutaraldehyde. The morphological characteristics of the bacteria before and after Pb treatment were observed by SEM after ethanol gradient dehydration, critical point drying and sputter-coating [21].

### 2.7. FTIR Analysis

The strains were inoculated in liquid medium with 0, 100 mg/L Pb^2+^ at 6% inoculum. Bacterial cultures and collection methods were the same as described above. The collected bacterial cells were freeze-dried overnight at −80 °C and then vacuum freeze-dried. The dried bacteria were first mixed with potassium bromide and then ground in an agate mortar at a ratio of 1/100 for the preparation of pellets. The resulting mixture was pressed with a pressure of 10 ton for 2 min. Fourier-transform infrared spectroscopy applied to pellet samples was used to analyze the changes in the functional groups on the surface of the bacterial cells [22].

## 3. Results and Discussion

### 3.1. Isolation, Screening, and Identification of Strains

After enrichment culture, isolation, and purification, 20 strains of bacteria were obtained, numbered D1~D20. Finally, a strain named D1 was rescreened from the twenty strains which could achieve 90% Pb removal in Pb^2+^ medium containing 200 mg/L.

**Figure 1:**
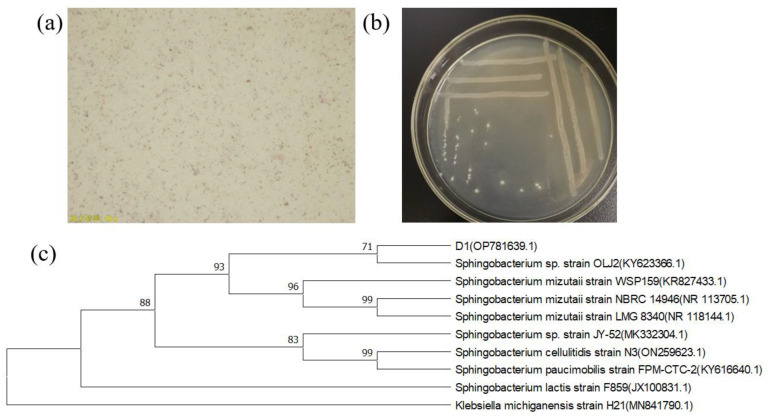
(**a**) Gram staining of strain D1; (**b**) morphological characteristics of strain D1; (**c**) phylogenetic tree of 16S rRNA.

Strain D1 colonies were characterized by a smooth, rounded, elevated, creamy white surface. The strain was rod-shaped, and the Gram stain was negative with flagella (Figure 1a,b).

The results of the 16S rRNA sequence comparison are shown in Figure 1c, and the D1 strain was tentatively identified as the *Sphingobacterium mizutaii* strain.

### 3.2. Effect of Environmental Factors on the Growth and Lead Removal Rate of Strain D1

**Figure 2:**
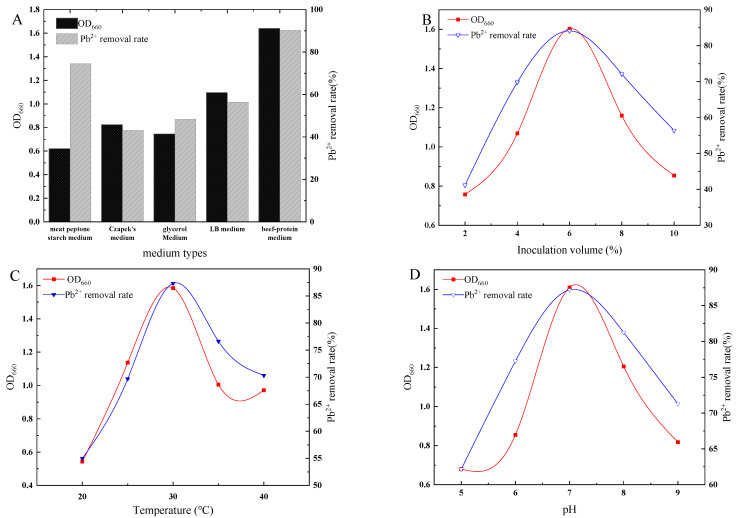

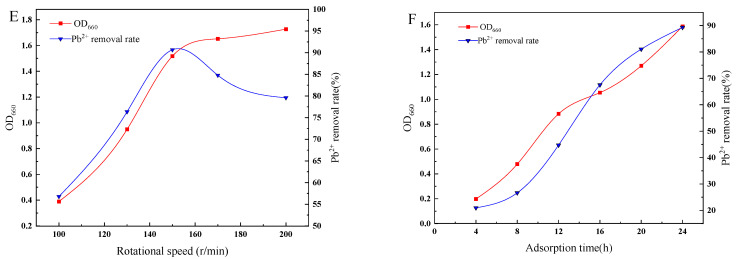
(**A**) Effect of medium type on the growth and lead removal rate of strain D1 (**B**) Effect of inoculum volume on the growth and lead removal rate of strain D1 (**C**) Effect of temperature on the growth and lead removal rate of strain D1 (**D**) Effect of pH on the growth and lead removal rate of strain D1 (**E**) Effect of rotational speed on the growth and lead removal rate of strain D1 (**F**) Effect of adsorption capacity on the growth and lead removal rate of strain D1.

The results of the effects of medium type, pH of the medium, inoculum volume, temperatures, rotational speed, and adsorption time on the growth and lead removal rate of strain D1 are illustrated in Figure 2.

Figure 2A shows that strain D1 had the most significant number of organisms, the best growth in the beef-protein medium, and the highest lead removal rate.

As shown in Figure 2B, with the gradual increase in the inoculum volume, the number of bacteria and the lead removal rate of strain D1 both increased and then decreased simultaneously. The optimum inoculum volume was 6%.

Temperature is one of the most critical environmental factors affecting microorganisms’ growth and metabolic activities. The maximum number of bacteria in the culture solution was found at a temperature of 30 °C, with a lead removal rate of 87.32% (Figure 2C).

The pH can affect the cell membrane structure of microorganisms and microbial enzyme activity. The maximum number of bacteria in the culture solution was found when the pH registered 7, the lead removal rate being 87% (Figure 2D).

With the regular increase in the rotational speed, the number of strains of D1 increased and then decreased, with the same trend of lead removal (Figure 2E). The maximum lead removal rate of D1 was achieved when the rotational speed was 150 r/min, with the lead removal rate as high as 90%.

The adsorption capacity of the strains varied with time. With the increase in the adsorption time, the number of strains and the lead removal rate of strain D1 showed an increasing trend (Figure 2F). After the adsorption time reached 24 h, the removal rate of lead by the strain gradually reached 89%.

### 3.3. Analysis of Orthogonal Test

Various factors affect the lead removal efficiency of strains, and the orthogonal test was designed to investigate the optimal growth conditions of strain D1. The results of the orthogonal test are shown in Table 2. From the definition of R (R represents the difference between the maximum and minimum values of the average index at each level of a factor), it is clear that the larger the extreme difference value of a factor, the greater the influence of the factor on the test results. Comparing the ranges in Table 2, we can determine the order of influence of each factor on the growth of strain D1, which is rotational speed > temperature > inoculation amount > pH value. The main effect values of each single factor were quantified using K values, where a larger K value indicates a greater impact on the experimental results. By comparing the average K values of each factor, we obtained the optimal growth conditions for strain D1, which were pH 7, 6% inoculation amount, 35 °C temperature, and 150 r/min rotational speed. Under these conditions, the strain could achieve a lead removal rate of up to 91%.

### 3.4. Mechanistic Study on the Removal of Lead by Strain D1

#### 3.4.1. SEM-EDS Analysis of D1 Strains Grown in the Lead and Lead-Free Environments

**Figure 3:**
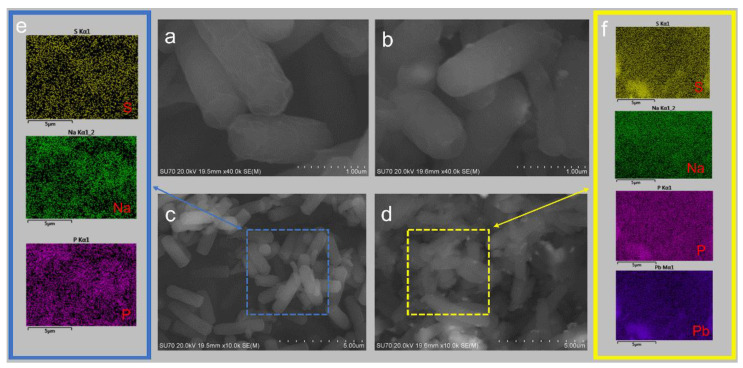
(**a**) SEM of strain D1 before adsorption (1 μm), (**b**) SEM of strain D1 after adsorption (1 μm), (**c**) SEM of strain D1 before adsor ption (5 μm), (**d**) SEM of strain D1 after adsorption (5 μm), (**e**) elemental composition of strain D1 before adsorption, (**f**) elemental composition of strain D1 after adsorption.

Figure 3 shows the visual appearance of the D1 cells before and after they were exposed to Pb^2+^. From Figure 3a, the surface of strain D1 grown in the lead-free environment was smooth and full, with intact bacterial morphology and a relatively regular distribution of filaments on the bacterial surface. By comparison, the surface of strain D1 grown in the lead environment (Figure 3b) changed from smooth to rough. The outline of bacterial cells became blurred with obvious deformation, and there were particles attached to the surface of the bacteria. This indicated the presence of cell surface adsorption of Pb^2+^ by strain D1. The elements were uniformly distributed on the cell surface before strain D1 absorbed the heavy metal Pb^2+^ (Figure 3e), and no Pb^2+^ was detected. After Pb^2+^ adsorption (Figure 3f), Pb^2+^ ions were detected on the surface of the strain. The results further confirmed that a certain amount of Pb^2+^ was indeed trapped on the surface of the cell.

**Figure 4:**
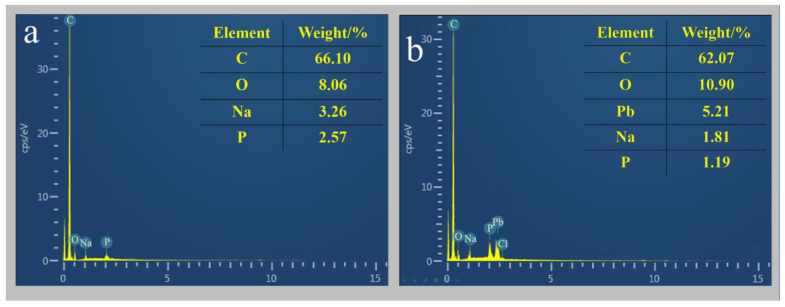
(**a**) Energy spectrum analysis of strain D1 before adding lead; (**b**) energy spectrum analysis of strain D1 after adding lead.

The results of the EDS analysis of strain D1 before and after the adsorption of heavy metal Pb^2+^ are shown in Figure 4. Comparing the energy spectrum images before and after adsorption, it can be seen that significant Pb^2+^ peaks appear after the adsorption of Pb^2+^. After the adsorption of Pb^2+^, the content of Pb^2+^ elements on the surface of strains reached third place, after C and O elements.

#### 3.4.2. FTIR Spectra of the Screened Strains before and after Pb^2+^ Adsorption

**Figure 5:**
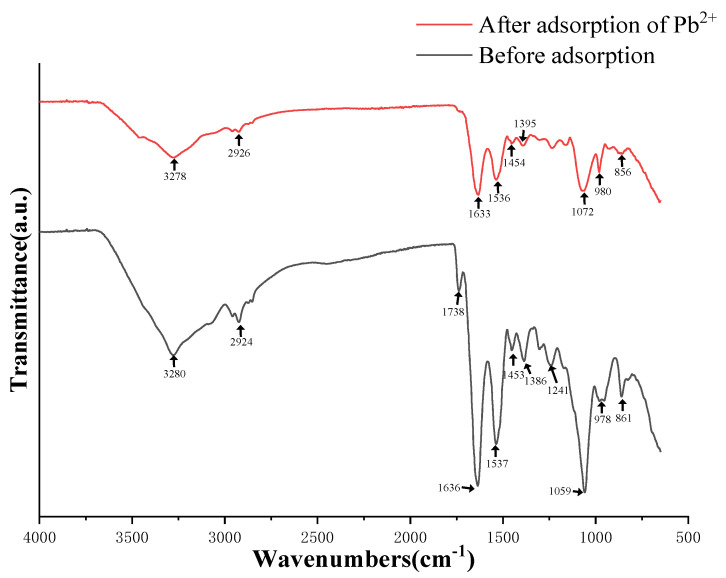
Infrared spectra of D1 before and after the biosorption of Pb^2+^.

The FTIR spectra of strain D1 before and after Pb adsorption are shown in Figure 5. The broad absorption peak at 3280 cm^−1^ represents the vibration of -OH and -NH [23]. The peak at 2924 cm^−1^ represents the C-H stretching vibration of phospholipid fatty acids in the cell membrane [24]. The absorption peak at 1738 cm^−1^ represents the stretching vibration of C-O [25]. The strong absorption peak at 1636 cm^−1^ is the amide I band caused by the C=O stretching vibration of the amide group [22]. The absorption peak at 1537 cm^−1^ is the amide II band caused by the N-H bending vibration [26]. The absorption peak at 1453 cm^−1^ is the -CH in-plane bending vibration in the peptide chain [27]. The absorption peak at 1241 cm^−1^ is the protein amide III band caused by the stretching vibration of -CN [28]. The strong absorption peak at 1059 cm^−1^ is caused by the vibration of C-O or P-O in polysaccharides [29]. 

### 3.5. Discussion

#### 3.5.1. The Effect of Culture Conditions on the Growth and Pb^2+^ Adsorption Effect of the Strains

The adsorption effect of microorganisms on heavy metals is strongly influenced by their species, growth, and culture environment. To find out the optimal conditions for lead adsorption by strain D1, the effects of pH, temperature, and adsorption time on the Pb^2+^ adsorption effect were investigated in this paper. The environmental pH can affect the growth of strains, the morphology of metal-binding sites, and the existence of heavy metals in the solution. Thus, it affects the adsorption effect of the strain on Pb^2+^. Metal binding sites are saturated with additional protons when the pH value is low [30]. At such a pH value, hydrogen ions increase the positive charge on the adsorbent surface. The attraction between the free state of metal ions and the adsorbent decreases. At a higher pH, Pb ions in the medium can act as protons, thus producing hydroxyl metal complexes under certain conditions. While some of these molecules, such as Ni and Cd, are soluble, Pb is not. This will reduce the amount of Pb^2+^ adsorbed by strains. In this study, neither pH levels below 7 or above 7 were conducive to bacterial growth and Pb^2+^ adsorption, and the best Pb^2+^ absorption was achieved when the pH was 7.

Temperature influences not only the ionization activity of the functional groups on the cell surface, but also the stability of the complexes formed by the functional groups and metal ions. Temperature affects the growth of the strain; it also reduces the affinity of the functional groups on the strain surface to Pb^2+^ and the activation energy required in the process of Pb^2+^ adsorption. The strain D1 in this study showed the highest growth and Pb^2+^ adsorption at 30 °C.

The adsorption time also affects the effect of Pb^2+^ adsorption of the strain. When the adsorption time is short, due to the rapid growth of strains, the heavy metal ions in the medium are gradually absorbed by the strains. With increasing adsorption time, the growth of strains consumes nutrients in the medium, making their biomass stable. The lack of nutrients and increased growth metabolites of strains can hurt the growth of strains and reduce the amount of Pb^2+^ adsorbed. In this study, the adsorption of lead by strain D1 increased gradually with the increase in adsorption time and reached 89% at 24 h.

Many environmental factors, such as temperature, pH value, medium types, and inorganic salt types can influence microorganisms’ growth capacity. In order to obtain the trend of each factor’s influence on the growth of the strain and find the optimal combination of parameters, variance analysis and range analysis were conducted on the orthogonal experimental results. This study found that the magnitude of the impact of each individual factor on the growth of the D1 strain was: rotational speed > temperature > inoculation amount > pH value. Combining the results of single factor experiments, the optimal growth conditions for strain D1 were determined to be pH 7, an inoculation amount of 6%, a temperature of 35 °C, and a rotational speed of 150 r/min.

#### 3.5.2. Study of Pb^2+^ Adsorption Mechanism by Strain D1

The mechanisms of microbial remediation of lead contamination include extracellular adsorption, intracellular accumulation, and transformation. Extracellular adsorption relies mainly on the binding of heavy metal ions by extracellular polysaccharides covered by the cell surface and chemical groups on the cell wall [31]. This stage does not require energy consumption and is characterized by a short adsorption time and fast adsorption speed. Intracellular accumulation and transformation is an active process; heavy metal ions pass through the cell wall and membrane into the cell, and are transported by microorganisms through compartmentalization to areas of slow metabolism [32], such as vesicles, or the heavy metal ions bound to specific proteins are transformed into a lower toxicity binding state. However, this process generally proceeds slowly and requires energy consumption [33]. The SEM observation of the morphology of the strain before and after Pb^2+^ adsorption showed that the strain D1 under Pb-containing culture was significantly deformed. The precipitation of white particles on the surface of the bacterium might be the result of the stress effect caused by Pb^2+^ on the bacterium. It induced strains to produce secretions to precipitate Pb^2+^ on the cell surface to reduce its toxicity. This suggests that extracellular precipitation is probably a mechanism of Pb^2+^ fixation by the bacterium and an essential reason for its adsorption of Pb^2+^. The result, that Pb^2+^ ions were detected on the surface of the strain after Pb^2+^ adsorption, further confirmed that a certain amount of Pb^2+^ was indeed trapped on the surface of the cells.

The EDS analysis of the strain elements showed that the lead element content increased substantially in the strain after Pb^2+^ adsorption compared to before Pb^2+^ adsorption. This corroborates with the SEM results.

When exposed to heavy metal stress in the environment, some microorganisms utilize specific proteins [34], functional groups on their surface [35], or secretions from their metabolic processes [36,37] to chelate heavy metal ions or induce precipitation to reduce their toxicity [38]. Previous studies have shown that microorganisms’ sequestration of heavy metals may be a complex process involving multiple reactions [35]. Fourier transform infrared spectroscopy (FTIR) analysis of functional groups revealed that, compared to the control without Pb treatment, the presence of Pb significantly affected -OH, -NH, and C-H in phospholipid fatty acids in the cell membrane, protein amides, -CH in the peptide chain, and C-O or P-O in polysaccharides. This resulted in an apparent decrease or shift in the absorption peaks representing these functional groups, indicating the involvement of multiple functional groups in the adsorption of Pb, which is consistent with previous research findings [39,40].

## 4. Conclusions

A strain with high tolerance to lead was isolated and purified from the residual sludge of factory-treated water in this study. The lead removal rate could reach 91% in a 200 mg/L lead culture solution. Morphological observations and molecular biological methods identified the strain as the *Sphingobacterium mizutaii* strain.

The effects of medium type, temperature, pH, and inoculum on the growth and lead removal rate of strain D1 were investigated. The results of orthogonal experiments showed that the most suitable medium for Pb^2+^ removal by strain D1 was the beef-protein medium, with pH 7, inoculum amount 6%, and temperature 35 °C.

Strain D1 showed good adsorption efficiency for Pb, and functional groups on the bacterial cell surface such as -OH, -NH, -CH, -C-O, -P-O, and amide groups were involved in the extracellular adsorption process of Pb.

The screened strain D1 has a strong ability to remove Pb^2+^. Based on the research results above, this strain has great application prospects in the field of microbial remediation of heavy metal pollution, and can be wielded for the bioremediation of low mass concentration of lead pollution.

## Figures and Tables

**Table 1:** Factors and levels of the orthogonal test.

Level	Factor			
	pH	Inoculation Volume (%)	Temperature (°C)	Rotational Speed (r/min)
1	6	4	25	130
2	7	6	30	150
3	8	8	35	170

**Table 2:** Results of the orthogonal test for strain D1.

Processing Number	pH	Inoculation Volume (%)	Temperature (°C)	Rotational Speed (r/min)	OD_660_
1	6	4	25	130	0.82
2	6	6	30	150	0.87
3	6	8	35	170	0.86
4	7	4	30	170	0.84
5	7	6	35	130	0.87
6	7	8	25	150	0.85
7	8	4	35	150	0.86
8	8	6	25	170	0.84
9	8	8	30	130	0.78
K_1_	2.55	2.52	2.51	2.47	
K_2_	2.56	2.58	2.49	2.58	
K_3_	2.48	2.49	2.59	2.54	
K_1_/3	0.850	0.840	0.837	0.823	
K_2_/3	0.853	0.860	0.830	0.860	
K_3_/3	0.827	0.830	0.863	0.847	
R	0.027	0.030	0.033	0.037	

## Data Availability

The data that support the findings of this study are available from the corresponding author, L.B., upon reasonable request.
